# Gastric diverticulum - ‘Double pylorus appearance’

**DOI:** 10.4103/0972-9941.15246

**Published:** 2005-03

**Authors:** Kaushik Bhattacharya

**Affiliations:** Department of Surgery, Sri Ramachandra Medical College and Research Institute, Deemed University, Chennai - 600116, India

**Figure 1:**
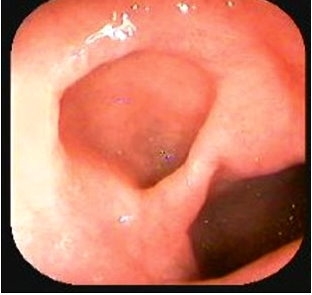
Double pylorus appearence of gastric diverticulum

A 40-year-old female presented with recurrent upper abdominal pain immediately after taking food. Upper gastrointestinal endoscopy revealed a gastric diverticulum (GD) measuring 3 cm × 3 cm on the anterior wall near the pylorus presenting as a ‘double pylorus’ (Figure 1). GD are one of the most rare and controversial gastrointestinal pathologies; very few cases are reported in the literature. Usually they are asymptomatic, single, saccular in shape, 1-4 cm in size and predominantly encountered in the 5^th^ or 6^th^ decade of life. Surgical intervention is required only in symptomatic patients and complicated cases. The most common diagnostic dilemma is that a GD simulates left adrenal massradiologically.[1] A GD should be differentiated from a gastroduodenal fistula, or a double-channel pylorus, which is caused by a penetrating ulcer in the distal antrum that erodes directly into the base of the duodenal cap or into the bulb. In this condition two channels communicate between the antrum and pylorus:the true pyloric canal and the fistula.
